# Chronic Lymphocytic Leukemia and Myelofibrosis

**DOI:** 10.1155/2018/7426739

**Published:** 2018-08-08

**Authors:** Fares Darawshy, Arieh Ben-Yehuda, Karine Atlan, Deborah Rund

**Affiliations:** ^1^Internal Medicine Division, Internal Medicine C Department, Hebrew University-Hadassah Medical Organization, Jerusalem, Israel; ^2^The Pathology Institute, Hebrew University-Hadassah Medical Organization, Jerusalem, Israel; ^3^Hematology Department, Hebrew University-Hadassah Medical Organization, Jerusalem, Israel

## Abstract

**Background:**

Chronic lymphocytic lymphoma (CLL) can be associated with several malignancies, but rarely with myelofibrosis. Only isolated case reports in the literature described the association between CLL and primary myelofibrosis (PMF) in the same patient.

**Objectives:**

We describe a case of CLL characterized by the development of PMF and a review of literature.

**Methods:**

We describe an 86-year-old female diagnosed as having CLL and followed by the development of splenomegaly and progressively rising LDH levels 27 months later. A bone marrow biopsy was consistent with the diagnosis of PMF, with positive JAK-2 V617F mutation. We also review the clinical and molecular characteristics of patients with CLL and PMF.

**Results:**

Patients with CLL and PMF are usually older. A lead diagnosis of CLL harbored by PMF is the most common clinical course, although concomitant diseases may occur in 31.7% of patients. JAK-2 V617F mutation can be found in 48.7% of patients.

**Conclusion:**

This case reported here constitutes an unusual situation of CLL characterized by the development of PMF. Etiologic and pathogenic associations—the role of *t* (1; 6) and JAK-2 V617F mutation—are discussed.

## 1. Introduction

Chronic lymphocytic leukemia (CLL) is a mature B-cell low-grade lymphoproliferative malignancy, characterized by monoclonal B lymphocytes expressing the CD5 antigen. This malignancy stems from mature lymphocytes in the germinal center of human lymph nodes, and bone marrow involvement in CLL is considered to be secondary. Chromosomal abnormalities that can be found in cytogenetic analysis include trisomy 12 and abnormalities in chromosome 13 [1, 2]. Primary myelofibrosis (PMF) is a disorder of a multipotent hematopoietic progenitor cell that causes clonal myeloproliferation accompanied by reactive bone marrow fibrosis and extramedullary hematopoiesis [3]. JAK-2 mutation can be found in 50% of PMF patients [4], and chromosomal abnormalities include trisomy 8, trisomy 9, and deletion 13, 9, or 20.

CLL has been associated with several malignancies, including transformation to diffuse large B-cell lymphoma and solid neoplasms [5], and any bone marrow involvement in CLL is assumed to be secondary. Usually, CLL is not associated with primary or secondary bone marrow fibrosis. The association between CLL and PMF and other myeloproliferative neoplasms (MPN) is unusual and has been reported [6–16]. Despite the growing number of reported patients with CLL and myeloproliferative disorders, the incidence of concomitant CLL and PMF is underreported and the clinical, molecular, and prognostic data are still lacking. The purpose of this report is to describe a patient diagnosed with CLL, and her clinical course was characterized by the development of myelofibrosis. A review of the literature and possible etiologic and pathogenic associations are discussed.

## 2. Case Report

An 86-year-old female, with a history of hypertension, type 2 diabetes mellitus, hiatus hernia, and diverticulosis, attended the hematology clinic in February 2003 for evaluation of lymphocytosis. Physical examination revealed neither lymphadenopathy nor organomegaly. The hemoglobin concentration was 11.4 g/dL, leukocyte count was 16.1 × 10^9^/L with 60% lymphocytes, mean corpuscular volume (MCV) 88.4 fL, and platelet count 332 × 10^9^/L. LDH was 569 units, mild IgG paraproteinemia (1130 mg/dL). Phenotypic analysis of blood lymphocytes by flow cytometry revealed CD5/19+ coexpression of 78% of the lymphocytes. No bone marrow biopsy was done. On abdominal ultrasound from June 2002, the spleen length was 9 cm. All these findings were suggestive for the diagnosis of CLL, Rai stage 0/Binet stage A.

**Figure 1:**
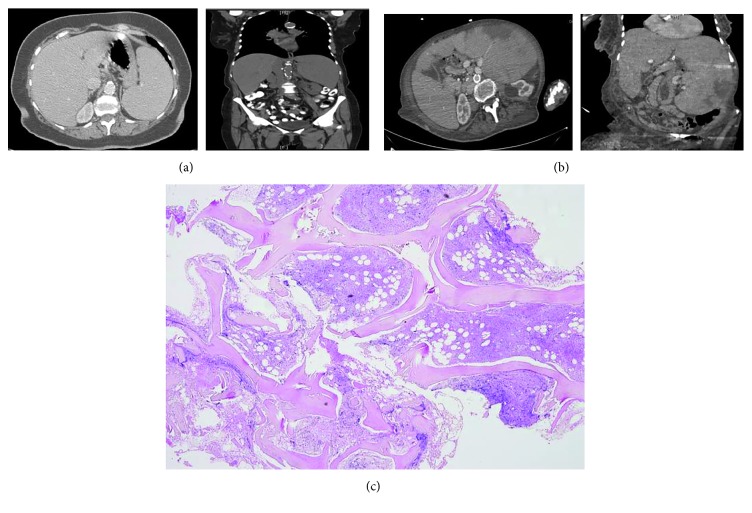
CT scan showing (a) 15 cm splenomegaly after CLL diagnosis in 2006. (b) CT scan in 2013 showing increased massive splenomegaly (28 cm) with splenic infarct and hepatomegaly, after PMF diagnosis. (c) Bone marrow biopsy showing hypercellular bone marrow with fibrosis, consistent with the diagnosis of myelofibrosis.

**Figure 2:**
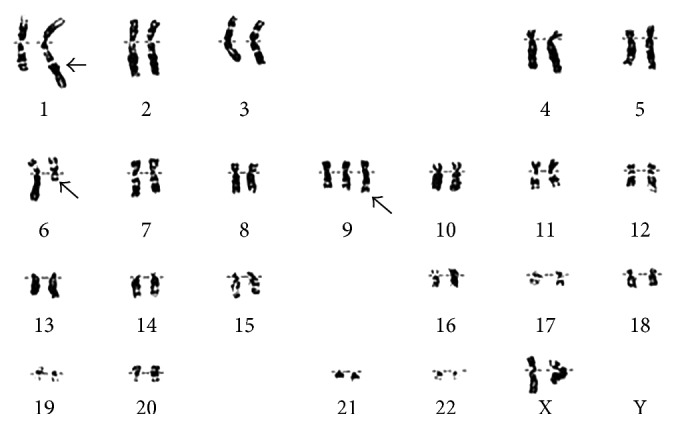
Karyotype showing trisomy 9, *t* (1; 6).

The patient was followed up for 27 months, during which progressive disproportionate splenomegaly (15 cm) with progressive rise in serum LDH (999 units) developed. In addition, the patient complained of anorexia and 30 kg weight loss. Physical examination revealed an enlarged spleen approximately 6 cm below the costal margin, but no palpable lymphadenopathy or hepatomegaly. Complete blood count showed hemoglobin concentration of 11.3 g/dL, leukocyte count of 19.8 × 10^9^/L with 58% lymphocytes, and platelet count of 240 × 10^9^/L. Peripheral blood smear revealed teardrop-shaped and nucleated red blood cells and immature cells of the myeloid lineage. Bone marrow biopsy revealed a hypercellular bone marrow with increased reticulin stain and fibrosis (Figure 1). A karyotype from the bone marrow showed chromosomal abnormalities of trisomy 9 (+9) and *t* (1; 6) (Figure 2). All findings were compatible with the diagnosis of myelofibrosis. The presence of the JAK-2 mutation was not examined at the time of diagnosis. Cytometric analysis of blood lymphocytes showed a majority of B cells (77% CD19+, 80% CD20+, and 42% CD22+). The patient was lost to follow-up for 2 years and admitted to the internal medicine department three times, two years later. Patient's last admission was due to anorexia, weight loss, dysphagia, and recurrent aspirations. Complete blood count showed normocytic anemia and stable lymphocytosis. Peripheral blood smear was compatible with bone marrow fibrosis. Imaging studies showed massive splenomegaly (Figure 1, about 28 cm on CT scan). With a diagnosis of suspected PMF presenting with massive splenomegaly, the patient was advised to be treated with ruxolitinib, a JAK-2 inhibitor. The patient refused to take any treatment and died due to infection. After her death, JAK-2 V617F mutation analysis was positive in the bone marrow biopsy that revealed bone marrow fibrosis, assuring the diagnosis of PMF.

## 3. Discussion

A variety of lymphoproliferative diseases can cause secondary bone marrow fibrosis, including Hodgkin's disease, multiple myeloma, hairy cell leukemia, and non-Hodgkin's lymphoma. In the patient described above, a diagnosis of myelofibrosis was made subsequent to CLL.

In the above reported patient, a diagnosis of CLL preceded PMF diagnosis by 27 months, based on phenotypic analysis and complete blood count only. Although bone marrow biopsy was not done at the time of CLL diagnosis, patient had no symptoms, signs, or laboratory criteria suggestive for myelofibrosis. The bone marrow biopsy done later, together with JAK-2 V617F mutation, confirms the diagnosis of PMF.

The association or coexistence of CLL with PMF in the same host is extremely unusual but was reported previously. Three cases of rapidly developing myelofibrosis after the diagnosis of CLL [5, 6, 12, 13] were reported, in addition to reports describing the coexistence of CLL and myelofibrosis [7, 8, 14]. In addition, a growing evidence from retrospective reviews revealed a clear association between lymphoproliferative disease and MPN, especially CLL and MPN. A retrospective review reported 46 cases suffering from concomitant CLL and myeloproliferative disorders, 4 of whom were PMF [10]. There was no association between the development of the myeloproliferative malignancy and the chemotherapy given for CLL. In addition, the course of CLL was not affected by the treatment of PMF and vice versa. Todisco et al. [15] retrospectively examined the clinical and biological characteristics of 13 patients with CLL and MPN, 8 of them with PMF which occurred in 1 patient who was followed and treated for CLL and in one patient concomitantly with CLL. JAK-2 mutation was found in 3 patients with CLL/PMF. These findings suggest that CLL and PMF pathological etiology and process and cooccurrence might not be random. Our patient's clinical course and findings are compatible with that reported by Todisco et al. Marchetti et al. [16] reviewed data from 50 papers reporting 214 individuals harboring both MPN and lymphoproliferative disease. Among 50 patients with myelofibrosis, 23 harbored CLL—7 were synchronous, 12 cases with prior CLL, and 4 with prior myelofibrosis. CLL was the most common lymphoproliferative disease particularly among patients with lymphoproliferative disease preceding MPN, and most of subsequent MPN developed by CLL patients were PMF. The median time between CLL and MPN diagnosis was 72 months.

Despite the number of reported patients with CLL and PMF getting larger, the clinical, molecular, and prognostic characteristics are still lacking. Tables 1 and 2 show the major clinical and molecular characteristics of available reported cases and series with CLL and PMF diagnosis in the same patient. Overall, 41 cases were reported, and average age was 61.75 years. Although some data are lacking, most of the reported patients with available data were males, and a finding by Marchetti et al. also reported that CLL and PMF can present concomitantly (in 13/41 (31.7%) of cases) or CLL can precede PMF diagnosis, with lead time varying between 2.3 and 13.6 years. Analysis of survival is limited due to lack of data, but previous reports [16] did not show any significant difference from the overall population except for higher age at MPN diagnosis. In addition, molecular data available are very limited, but it is worth mentioning that JAK-2 V617F mutation was present in 20/41 (48.7%) of reported cases, compared to 37.5% reported by Todisco et al.

The exact mechanism in which myelofibrosis develops in the presence of CLL is also still unclear. Kimura et al. reported a case of CLL with secondary myelofibrosis and confirmed the role of interleukin-1 alpha (IL-1*α*) in the pathogenesis, stating that CLL cells secrete IL-1 which stimulates the growth of fibroblasts, causing bone marrow fibrosis in vitro [11]. Several pathogenic mechanisms were suggested to explain the association between CLL and PMF, including independent proliferations of two separate clonal cells causing two unrelated diseases, chromosomal or molecular abnormality in a multipotent cell causing bilineage clonal proliferation and incidental association [6, 8].

In our case, the diagnosis of PMF was confirmed by the histological method. In our opinion, in the reported patient, CLL preceded the development of PMF, and JAK-2 V617F mutation contributed to PMF development. This hypothesis is supported by results of previous studies [10, 15, 16] showing that CLL is the most common lymphoproliferative disease preceding PMF, along with the development of JAK-2 V617F mutation which is known to increase the risk for lymphoma [17, 18]. Our patient did not have any therapy for CLL or other types of cancer, deferring therapy, and cancer-related myelofibrosis. Unfortunately, JAK-2 mutation and karyotype were not done at time of CLL diagnosis, but it was done later showing JAK-2 V617F mutation, trisomy 9 which was known to be associated with PMF, and *t* (1; 6) which was reported by L. Michaux et al. to be a novel cytogenetic aberration exclusively found in unmutated CLL [19]. This translocation was found to be associated with an aggressive clinical course but had no known association to myelofibrosis. We do notice the coexistence of *t* (1; 6) and trisomy 9, chromosomal abnormalities that appear in CLL and PMF, but no clear association can be found between both. We do suggest further evaluation of the role of *t* (1; 6) in bone marrow fibrosis in future studies.

## 4. Conclusion

In this case report, we reported a patient in whom a diagnosis of CLL and myelofibrosis was made. No clear association was found between these two diseases in past reports. Older patients tend to develop CLL and PMF, with the most common clinical course of CLL preceding PMF diagnosis, and nearly 50% of patients have JAK-2 V617F mutation. Unfortunately, we could not find any new molecular or chromosomal abnormalities that explain the development or coexistence of myelofibrosis in CLL, but we do suggest the role of JAK-2 V617F mutation and *t* (1; 6) as main molecular abnormalities.

## Figures and Tables

**Table 1:** Clinical characteristics of reported patients with CLL and PMF.

Report	Patient's number	Age at CLL diagnosis, mean (years)	Sex, males/females (% males)	Time between CLL and PMF Dx, median (years)	Chemotherapy, *n* (%)	Simultaneous diagnosis of CLL and myelofibrosis, *n* (%)	Death after myelofibrosis diagnosis, *n* (%)	Survival, median (years)
Lennard et al. [6]	1	42	0/1 (0)	6.3	1 (100)	0 (0)	1 (100)	0.45
Nieto et al. [7]	1	70	0/1 (0)	2.3	Not given	0 (0)	Not available	Not available
Palta et al. [8]	1	60	1/0 (100)	0	Prednisolone	1 (100)	Not available	Not available
Burgstaller et al. [9]	1	61	0/1 (0)	0	Lenalidomide	1 (100)	No	Not available
Laurenti et al. [11]	4	69.75	4/0 (100)	Not available	1 (25)	2 (50)	Not available	Not available
Salama et al. [14]	2	68	2/0 (100)	4.5 (0–9)	Hydroxyurea	1 (50)	No	Not available
Todisco et al. [15]	8	60.3	Not available	6.05 (0–13.6)	5 (62.5)	1 (12.5)	4 (50)	8.9 (0.7–15.7)
Marchetti et al. [16]	23	66.5 (26–81)	Not available	6	Not available	7 (30.4)	Not available	Not available

**Table 2:** Molecular characteristics of reported patients with CLL and PMF.

Report	Karyotype at CLL diagnosis	IGHV mutation, *n* (%)	JAK-2 mutation, *n* (%)	Karyotype at myelofibrosis diagnosis
Lennard et al. [6]	Not available	Not available	Not available	Not available
Nieto et al. [7]	Not available	Not available	Not available	Not available
Palta et al. [8]	Not available	Not available	Negative	Not available
Burgstaller et al. [9]	Normal	Not available	Negative	Normal
Laurenti et al. [10]	Not available	1 (25)	2 (50)	Not available
Salama et al. [14]	Not available	Negative	Negative	Not available
Todisco et al. [15]	Normal (50%)	4 (50)	3 (37.5)	Del 7 (12.5)
	*t* (3; 12) (12.5%)			Del 8q21, del 20q11 (12.5)
	Del 20q11 (12.5%)			Del 5q, del 13q (12.5)
	Del 5q, del 13q (12.5%)			*t* (3; 12) (12.5)
	Not available (12.5%)			
Marchetti et al. [16]	Not available	Not available	15 (65.2)	Not available
